# Safe medication management in specialized home healthcare - an observational study

**DOI:** 10.1186/s12913-017-2556-x

**Published:** 2017-08-24

**Authors:** Marléne Lindblad, Maria Flink, Mirjam Ekstedt

**Affiliations:** 1 Royal Institute of Technology, School of Technology and Health, Stockholm, Sweden; 2 Department of Health Care Sciences, Ersta Sköndal University College, Stockholm, Sweden; 3 Department of Learning, Informatics, Management, and Ethics, Karolinska Institutet, C7, Tomtebodavägen 18a, S-17177 Stockholm, Sweden; 4 Department of Social Work, Karolinska University Hospital, Stockholm, Sweden; 5 School of Health and Caring Sciences, Faculty of Health and Life Sciences, Linnaeus University, Kalmar, Sweden

**Keywords:** Home healthcare, Medication management process, Medication administration, Medication errors, Patient safety, Resilience

## Abstract

**Background:**

Medication management is a complex, error-prone process. The aim of this study was to explore what constitutes the complexity of the medication management process (MMP) in specialized home healthcare and how healthcare professionals handle this complexity. The study is theoretically based in resilience engineering.

**Method:**

Data were collected during the MMP at three specialized home healthcare units in Sweden using two strategies: observation of workplaces and shadowing RNs in everyday work, including interviews. Transcribed material was analysed using grounded theory.

**Results:**

The MMP in home healthcare was dynamic and complex with unclear boundaries of responsibilities, inadequate information systems and fluctuating work conditions. Healthcare professionals adapted their everyday clinical work by sharing responsibility and simultaneously being authoritative and preserving patients’ active participation, autonomy and integrity. To promote a safe MMP, healthcare professionals constantly re-prioritized goals, handled gaps in communication and information transmission at a distance by creating new bridging solutions. Trade-offs and workarounds were necessary elements, but also posed a threat to patient safety, as these interim solutions were not systematically evaluated or devised learning strategies.

**Conclusions:**

To manage a safe medication process in home healthcare, healthcare professionals need to adapt to fluctuating conditions and create bridging strategies through multiple parallel activities distributed over time, space and actors. The healthcare professionals’ strategies could be integrated in continuous learning, while preserving boundaries of safety, instead of being more or less interim solutions. Patients’ and family caregivers’ as active partners in the MMP may be an underestimated resource for a resilient home healthcare.

## Background

Medication therapy is one of the most pervasive interventions in the health system. [1, 2]. Medication management is a complex, error-prone process [3, 4] which in Sweden includes the following steps: prescription, preparation, administration, requisition, control (of narcotics) and storage of drugs [5]. Mistakes during the medication management process, i.e., medication errors, are well-known threats to patient safety in both hospital [6] and home care settings [7, 8]. Medication errors could have serious implications for patients’ well-being, morbidity and mortality, including costs for the healthcare system and society [9].

Recent improvements in medical knowledge and technology have made it possible to transfer administration of potent drugs and the use of complex technology into patient homes [10], making home healthcare with advanced health interventions a growing arena [7, 11, 12]. Specialized home healthcare units are an expanding form of the traditional palliative home healthcare. Patients with chronic diseases have the possibility to choose this kind of care when basic home healthcare is insufficient, as an alternative to hospital care [13].

A high level of patient safety is a basic requirement in all healthcare organizations [14, 15]. Traditionally, patient safety work relies heavily on standardization, such as development of guidelines, education and training [16, 17]. Still, medication errors are common [18, 19]. As one cannot control complex processes through standardization or linear thinking, we should consider new ways of handling gaps or patient safety risks.

### Theoretical framework

Modern patient safety research is based on a dynamic and systemic approach, in which all parts of a system and their internal relationships are of interest, rather than individual components [17, 20]. Resilience engineering has attracted increased attention during the last decade, as a proactive approach to manage safety in complex socio-technical systems, in contrast to the traditional focus on failure and accidents [21]. Resilience engineering represents the ability of the system to adjust its functioning prior to, during or following both expected and unexpected events, and thereby sustain required strategies under varying conditions [20, 22]. In this perspective the basis for safety improvements involves understanding how daily work is actually done. The actual performance of work-as-done (WAD) is different from the idealized view of work-as-imagined (WAI). WAI does not consider the varying conditions under which everyday work is performed, whereas WAD reflects what the individuals actually have to deal with in a complex context [23]. Thus observations of how the healthcare professionals’ actually do to achieve success in their everyday work, and not merely avoid failures or adverse outcomes, was considered as an appropriate method to bring new insight into how high safety performance in the MMP in specialized home healthcare may be supported.

The overall aim of this study was to explore the MMP in specialized home healthcare from a resilience perspective. More specifically, we aimed to explore a) what constitutes the complexity of the MMP and b) how healthcare professionals handle the complexity of creating a safe MMP in everyday work.

## Method

### Design and study setting

This is an exploratory observational study. The observations, including interviews, were performed alongside healthcare professionals in three specialized home healthcare units, in order to acquire knowledge ‘from within’ [24]. This methodology enables studying naturally occurring events and processes in routine daily work, allowing for a deeper understanding of the dynamics within a given clinical context.

The three specialized home healthcare units are situated in an urban area of Sweden and were selected to cover different sociodemographic areas. These self-contained units are organizationally tied to local hospitals, tax-funded by the county council and each covers a limited geographical area. The units consist of ambulatory multidisciplinary teams, with RNs and physicians available 24 h a day, seven days a week, administering healthcare in patient homes. Patients have complicated medical and care needs and/or a need for advanced equipment (pain pump, respiratory equipment, etc.), and require advanced medication therapy with multiple “as needed” (PRN) medications. Common diagnoses of the patients are cancer, both curative and palliative phases, cardiovascular diseases, Chronic Obstructive Pulmonary Disease and neurological diseases. The patients commonly have multi-morbidity, polypharmacy and are often in contact with several healthcare specialists prescribing their drugs. Specialized home healthcare has the overall medical responsibility and all prescribed drugs are delivered by these units to the patients’ homes at no charge.

### Data collection

Data were collected between October 2013 and March 2014 using multiple techniques [24], including audio recordings, field notes, memo-writing and interviews with healthcare professionals involved in the observations.

The head of each department gave permission to conduct a part of the study at their unit. All healthcare professionals got both written and verbal information about the study procedures and were informed that they could decline from being observed or interviewed. The healthcare professionals informed the patients about the study and asked the patients beforehand if the researchers could observe the encounter.

The observations, performed by the first and last authors, focused on work processes involving medication management and two observation strategies were used: observation of ‘places’ and ‘shadowing’ [25]. Observation of places meant that the authors spent time observing different workstations in the units where preparation of drugs, care planning, meetings, documentation and handover of information occurred e.g., medicine rooms, offices, meeting rooms. Shadowing means “following people, wherever they are, whatever they are doing” [25] (p. 301). We primarily shadowed RNs who were actively involved in the MMP, as RNs have a major role in medication management [26] and are in a key position to identify and rectify errors before patients are affected [27]. Each day the manager decided which RN that we could shadow. In total, we shadowed 27 RNs’ during morning and evening shifts and their ordinary visits to patient homes observing their interactions with patients and other parties in the MMP. The intention was to interfere as little as possible with ongoing work. Unstructured interviews were conducted when the workload decreased, after observed situations or during transfers between places, to clarify and deepen the understanding of the MMP.

Data collection was structured in iterative cycles, followed by periods of transcription and initial coding, so that the analysis from one cycle informed the subsequent research questions, in accordance with grounded theory principles [28]. Initially, over a time period of six months, the observers spent time in the home healthcare units to get familiarised with organization, healthcare professionals, and routines. The initial phase was followed by three observation cycles; each cycle consisted of three days per unit. Each day consisted of 6–8 h observation. In total, observations were conducted during 27 days, nine days per unit.

### Data analysis

Field notes, memos and transcripts of audio-recorded communication during observations and interviews were analysed by all authors using constant comparative methods inspired by the constructivist version of grounded theory [28]. Initial line-by-line and incident-by-incident coding was conducted in a non-linear process and continuously compared based on similarities. New insights prompted us to look at data afresh and reconsider what was implicit in earlier coding. Codes were clustered and then theoretically abstracted and synthesized into four categories to describe the dynamic MMP, while preserving their specific connections to data, as illustrated by cases and quotations in the results. The codes, categories and relations between them were discussed by all authors frequently, until consensus was reached.

### Ethical considerations

This study was approved by the Regional Ethical Review Board, Stockholm (Ref: 1012/1384).

## Results

The analyses of the MMP identified four categories. The first category, *A dynamic MMP*, illustrates the complexity that stakeholders face in different stages of the MMP in specialized home healthcare. The other three categories, *Bridging unclear boundaries of responsibility*, *Creating interim solutions in inadequate information systems*, and *Coping with fluctuating work conditions*, illustrate the work-as-done for the healthcare professionals in the MMP, as well as the adaptations and prioritizations made to manage a safe MMP.

### A dynamic MMP

**Fig. 1:**
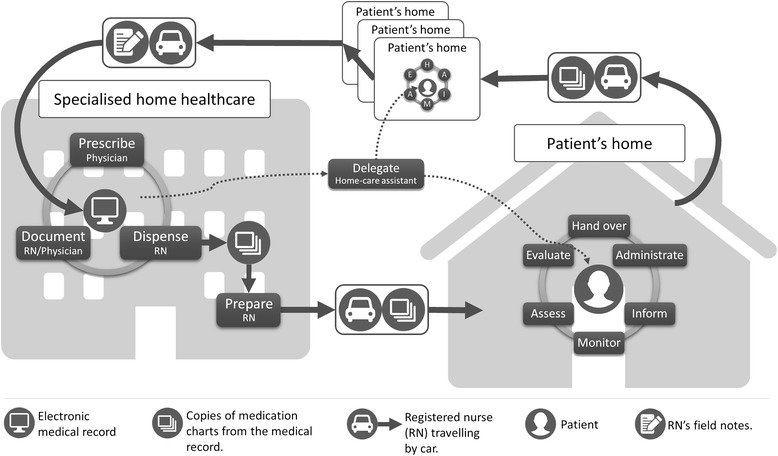
The medication management process in specialized home healthcare (© Lindblad, Flink, Ekstedt)

The MMP in specialized home healthcare was a dynamic process as illustrated in Fig. 1. Multiple parallel activities were distributed over time, space and actors – constituting a challenge for ensuring patient safety.

Prescription included both a technical component, when the physician prescribed medications in the electronic medical record (EMR), and a decision-making component, which was based on assessment of the patient’s medical needs. The physician was to print a copy of each patient’s medication list any time patient prescriptions were changed, to be stored in the unit’s emergency box in case the computer system crashed. The physicians also decided which medications would be distributed through pharmacies. These medications were delivered to the units in a multi-dose drug dispensing aid (Swedish: ApoDos®), packed in disposable bags, one for each administration, with information on patient data, contents, date and time for intake. These bags usually contained seven days’ worth of medication for each patient when delivered to the units. During dispensing, the day before delivery, a RN would check the bags from the pharmacies and dispense any other prescribed medications for each patient. At the units, a commonly used administration aid was a box (Swedish: dosett) with separate medication compartments for each day of the week and different times of day. Medications that could not be dispensed in these dispensing aids, e.g., liquids, inhalers and suppositories or PRN medications (e.g., opioids, benzodiazepines), were dispensed without original packaging or labels. The RN signed for the medications in the EMR and printed a copy of the current medication list to be stored along with the dispensed medications in a separate pouch for each patient, until the day of delivery. On the day of delivery, each RN in charge prepared the dispensed medication pouches for their scheduled patients, matching them against the current medication lists. During both dispensing and preparation the RNs needed to be aware of recent changes in prescriptions and manually change medications in the bags/boxes. During home visits, the RNs commonly handed over the medications to the patients and/or the family caregivers for administration, or delegated administration to unlicensed home care assistants (employed by a private or municipal home care provider). RNs administered medications in injection or infusion form. Monitoring, assessment, evaluation and provision of information about why, when and how the patients should take their medications were part of the RNs’ home visits. The EMR was not accessible in patient homes, and thus documentation took place at the unit after the scheduled home visits. Some RNs began making short memos after each visit. The written documentation was often supplemented with verbal information to the next shift.

### Bridging unclear boundaries of responsibility

Being in charge in a patient’s home constitutes a challenge: adapting to a shared responsibility and simultaneously being authoritative and preserving the patient’s active participation, autonomy and integrity.

Managing the MMP, from prescription to intake in the patient’s home, was a process in practice shared between the municipality, the patient, family caregivers and various care providers. Separate documentation systems and sparse information exchange challenged the RNs to find ways to communicate across organizational boundaries. A common bridging strategy was illustrated by the following case.

*A woman with multiple illnesses – of which one is diabetes – requires high-dose insulin injections several times a day. The RNs administer these injections and leave the patient’s home before the home care assistants come to prepare meals. Before home visits, the RNs usually discussed the patients they were going to visit with one another. On one such occasion, a RN mentioned that she was worried because she did not know how long it would take before the home care assistant could provide the woman with her meals. One RN shared that she used to give the woman a glass of milk before she left. They agreed that the RN should also write a note to the home care assistant and place it strategically in the patient’s home, informing about the times for insulin injections and the importance of coordinated mealtimes.*

Monitoring and evaluation of the therapeutic and unintended effects of medications was done from a distance. For patients with home visits scheduled once a week or less, such evaluation and the opportunity to discover possible misunderstandings was limited. Patients could not easily check with RNs if they had understood instructions correctly. Evaluation ahead of the physician’s medical decisions was mainly based on the RNs’ interpretations of patient self-reports. A common strategy to bridge the distance is illustrated by the following case:

*A man was to begin taking steroids. The nurse performed a home visit with the steroids and gave him information on how to take them. The steroids were to be dissolved in water before being taken. At the same visit the nurse advised the patient to rinse his mouth afterwards to avoid an unpleasant aftertaste. The following week, the patient felt even worse. The RN took the time to listen to the patient’s description and evaluate how he was taking the medication. It became clear that the man was rinsing his mouth with the steroid solution and then spitting it out.*

Patients often stored blister-packed PRN medications with similar names but completely different medical purposes in the same place. One example was the drug Oxynorm© (a opioid) commonly stored together with the drug Oxascand© (a benzodiazepine). The routines for checking patient needs of PRN medications before delivery often failed, meaning that the prescribed maximum dose was usually delivered every week. The RNs describe that the large amounts of stored drugs worried them, but that confiscating the drugs was difficult without interfering with the patient’s autonomy and integrity:

- *the thing about dispensing a lot of medication without knowing how much has already been issued... It’s really… quite… risky business (RN).*
- *Somewhere along the line they [the patients] have their own responsibilities. That’s the way it seems. You can’t take over everything – you don’t have the right to do that (RN).*

### Creating interim solutions in inadequate information systems

The shortcomings of IT systems contributed to gaps in communication and information transmission, making it necessary to constantly find new creative solutions.

The EMR was an essential tool, but had shortcomings leading to gaps in the MMP. For example, several system windows had to be open at the same time during dispensing. Hence, the healthcare professionals switched back and forth between windows for an overview of changes and current prescriptions. Patients frequently had multiple providers making changes in medications. Although the patient and/or family caregivers took a major responsibility in transferring information between different care providers, medication changes were easily lost, with implications for the patient, as illustrated in the following case.

*A woman with heart failure complained that she had difficulty breathing. The breathing problems had increased over the last month and symptoms worsened as soon as she moved or tried to do anything. When the RN reported back to the physician, he asked about the effects of the daily dose of intravenous diuretics prescribed the previous week. It transpired that the diuretic prescription had not been noticed by the RNs, thus the patient had not received the medication*. *The week after, the patient was hospitalized due to increased breathing problems.*

The EMR was unavailable in the patient’s home, so an interim solution was to leave a copy of the medication list there. RNs made handwritten notes and coloured highlights on the copy to clarify the prescribed medications to themselves and the patient. Patients made additional notes as a reminder and to facilitate their own understanding. The list copies were updated every week, or when the prescriptions were changed. This meant that the patients’ and RNs’ notes had to be re-written every week.

### Coping with fluctuating work conditions

The frequent and rapid variations during daily work constituted a challenge in prioritizing goals as well as in creating strategies to promote a safe MMP and translating it into learning.

The preparations before the home visits (1–1.5 h) were task-intense and required a high level of concentration, e.g., checking the pre-dispensed medication and correcting changes. The healthcare professionals should also participate in a team meeting with managers, an opportunity for safety debriefing where specific risks, medication errors and adverse drug events were discussed to increase the healthcare professionals’ awareness. Strategies to ameliorate risks or avoid errors were seldom addressed. This phase consisted largely of verbal information exchanged between the healthcare professionals.

Although the RNs thoroughly prepared for what could happen during the day, they constantly needed to adjust their work due to unpredictable staffing and patients’ conditions and needs. For example, RNs commonly had to switch patients although they had already prepared for their scheduled patients. These adjustments in turn generated workarounds and trade-offs on the part of the RNs, in order to be efficient and manage tasks on time. For example, RNs down-prioritized checking pre-dispensed medications and chose to rely on the colleagues who had done the pre-dispensing. They would skip team meetings, although they risked missing important information. They would choose not to read the EMR and prioritize face-to-face-communication with peers to get updates on patient status. Strategies to promote a safe MMP at an organizational level were part of the local medical guidelines. Each unit had developed about 30 medical guidelines. Other strategies included memo notes at strategic places in the unit, listing human resources or risks for the day, and having a quiet zone at the medical door and earplugs to wear during the dispensing phase. Memo notes were also found in the patients’ homes.

## Discussion

The complexity of the medication management process in home healthcare and the healthcare professionals’ strategies to handle this complexity was consistent with the resilience engineering perspective, which suggests that both risks and safety emerge from the same organizational processes in a variable and goal-conflicted dynamic system [29]. This study showed that the actual performance of the MMP was different from the idealized ‘map’ of how the MMP was intended, showing a linear process in the guidelines. Even though 30 medical guidelines were developed to safeguard the phases of the MMP (Fig. 1), trade-offs and workarounds from these guidelines were common in each studied setting. Standardization of work processes has been a legitimate part of the efforts to increase safety and streamline care [30]. The problem is that standardization of work processes is an imaginary ‘map’ of the intended workflow (WAI), which is rarely how work is done under ordinary conditions (WAD) [23]. Trade-off decisions were made as a result of conflicting goals [31], consistent with what is found in other studies [32, 33]. As emphasized in a review of RNs’ workarounds in emergency healthcare [16, 34], there are two sides to this coin. Workarounds can temporarily ‘fix’ a workflow hindrance or compensate for inadequate supplies (i.e., logistics, technology, expertise) [16] by adapting to circumstances and building resilient organisations. On the other hand, if interim solutions such as those developed to handle an inadequate information system become routine, they can effectively mask deficiencies in the organisation, undermine policies and rules, and thus become a threat to patient safety in the long run [16, 33]. Thus, *learning* from the adjustments made by healthcare professionals in everyday work that makes things succeed is crucial in order to engineer high-performance safety systems.

One of the key challenges identified in this study was how to bridge unclear boundaries of medical responsibility in the patients’ home and maintain a safe medication process in line with guidelines and rules, while at the same time preserving the patients’ autonomy and integrity. Swedish healthcare legislation has strengthened patients’ and family caregivers’ rights to take part in patient safety work [15] and to be active participants in all parts of their care [35]. The patient’s active role in sharing information and expert knowledge on their experience of the disease, with different members of the multi-professional team, may be an underestimated resource for a resilient healthcare system [36]. The patient’s responsibility to take the medications as prescribed and; monitor and report on needs, symptoms and treatment effects, may contribute to a raised awareness of potential errors. This study shows that the RNs had to prioritize between promoting patient participation in the MMP, good relations and communication with patients on one hand, and guidelines and rules on medication safety on the other hand, when these goals were contradictory. This raises ethical dilemmas, since a safe MMP in specialized home healthcare seems to require a balanced, shared decision-making between a professional and a patient [37, 38], while maintaining and safeguarding their relationship [39, 40]. A shared responsibility without a common foundation of understanding – and well-defined borders of authority – may thus encroach on safety.

This study highlights that allocating time for daily team meetings did not, per se, automatically induce learning. These meetings tended to be a ‘glance in the rear-view mirror’ with error reports, instead of a proactive forward-looking discussion about how errors and gaps might be anticipated and avoided. The potential to learn from both successful adaptations and failures is important for building resilient organizations [20]. Hallmarks in resilient healthcare systems are the ability to reflect, to share experiences, and to learn. A reflective and shared understanding of possible solutions could have increased organizational learning [41]. Instead, the proactive face-to-face communication on safety issues occurred mainly during daily work. Earlier studies have shown that proactive communication is a way to minimize medication errors and promote patient safety [42]. However, the drawback is that such ‘ad hoc-communication’ is rarely documented in the EMR [43, 44]. This impedes horizontal learning within the team as well as vertical learning between managers and staff.

Healthcare professionals and patients constantly struggled to bridge the gaps of a deficient EMR system. In 2008, the Swedish National Board of Health and Welfare claimed that the premise of safe and secure home healthcare involves staff having continual access to the EMR, especially when a patient has multiple healthcare providers from different organizations [12]. However, these intentions are still far from realized in specialized home healthcare. Written notes on paper copies of the EMR – initially made to assist patients and home care assistants in administrating medications safely – became dangerous pitfalls. Outdated lists and unclear notes created uncertainty about the prescriptions’ reliability and what medications the patient was currently taking. The many shortcomings in the IT system were a source of frustration and powerlessness, giving rise to homespun solutions that were a threat to safety.

### Limitations

Our research has some limitations to consider. Firstly, the use of the observation method to explore participants’ intentions and thoughts is a potential source of bias. Unstructured observations of the communication as well as interviews were collected to address this issue. Further, to reduce observer bias, we used researcher triangulation by involving two or three researchers in all stages of data collection, analysis and interpretation of data. Secondly, the RNs may have changed their behaviour and their way of working because they were being observed. However, the observers spent a certain amount of time in these settings before data collection started, allowing the healthcare professionals to get used to having an observer in their proximity and get an understanding of the purpose of the project. Data was collected in three home healthcare settings with different sociodemographic characteristics, to capture different challenges in the MMP. It may be that the choice of setting, a single urban area of Sweden, limits the extent to which our findings may be transferred to rural settings or other regions. On the other hand, the complexity of the MMP and factors associated with errors in this process, as well as the healthcare professionals’ strategies to handle the fluctuating situations, are similar across different healthcare settings. Thus, we believe that these findings can, on an aggregated level, be applied in other settings.

## Conclusions

To manage a safe medication process in home healthcare, healthcare professionals needs to adapt to fluctuating conditions and create bridging strategies through trade-offs, workarounds and face-to-face-communication with peers. To improve patient safety in the MMP, the healthcare professionals’ strategies need to be integrated in continuous learning and safety work, instead of being more or less permanent interim solutions. Home healthcare organizations could strive to increase resilience in the MMP by allocating time for reflection to increase awareness and preparedness to handle complexity and fluctuating conditions in everyday work. Such learning sessions could also incorporate discussions on how to handle the boundaries between patients’ and family caregivers’ participation, autonomy and integrity.

## Abbreviations

EMR: Electronic Medical Record
MMP: Medication Management Process
PRN medications: “as needed” medications (pro re nata)
RN: Registered Nurse
WAD: Work-As-Done
WAI: Work-As-Imagined
